# The relationship between white matter integrity of superior longitudinal fascicle and cognitive functions in chronic schizophrenia

**DOI:** 10.1192/j.eurpsy.2022.1984

**Published:** 2022-09-01

**Authors:** E. Tyburski, P. Podwalski, M. Mak, A. Michalczyk, J. Kucharska-Mazur, J. Samochowiec, L. Sagan

**Affiliations:** 1 Pomeranian Medical University in Szczecin, Department Of Health Psychology, Szczecin, Poland; 2 Pomeranian Medical University, Department Of Psychiatry, Szczecin, Poland; 3 Pomeranian Medical University in Szczecin, Department Of Psychiatry, Szczecin, Poland; 4 Pomeranian Medical University, , Department Of Neurosurgery, Szczecin, Poland

**Keywords:** chronic schizophrenia, white matter integrity, Diffusion Tensor Imaging, cognitive functions

## Abstract

**Introduction:**

Some evidence suggests that disruption of integrity in the superior longitudinal fascicle (SLF) may influence cognitive functions in chronic schizophrenia (CS) but the results are inconclusive.

**Objectives:**

Using diffusion tensor imaging tractography, we investigated the differences in fiber integrity between patients with CS and healthy controls (HC) together with the relationship between fiber integrity and cognitive functions.

**Methods:**

Forty-two patients with CS and 32 HC took part in the study. Assessment of cognitive functions was performed using Measurement and Treatment Research to Improve Cognition in Schizophrenia.

**Results:**

showed group differences, left and right in fractional anisotropy (FA) and mean diffusivity (MD) of the SLF, where patients showed less integrity than controls. Patients performed worse attention/vigilance, working memory, verbal learning, visual learning, reasoning and problem solving, and social cognition tasks than HC. However, when premorbid IQ and level of education were controlled for, the differences were no longer statistically significant in verbal learning and social cognition. In patients with CS, a positive correlation was found between FA of the left SLF and attention/vigilance and working memory. Moreover, in this group there was a negative correlation between MD of the left and right SLF and working memory and social cognition.

**Conclusions:**

These findings provide evidence that SLF disruption appears in patients with CS and might account for impairment of cognitive functioning. This research was funded by the Polish Minister of Science and Higher Education’s program named “Regional Initiative of Excellence” number 002/RID/2018/2019 to the amount of 12 million PLN.

**Disclosure:**

No significant relationships.

